# Valorization of spent double substituted Co–Ni–Zn–Fe LDH wastewater nanoadsorbent as methanol electro-oxidation catalyst

**DOI:** 10.1038/s41598-022-23798-2

**Published:** 2022-11-11

**Authors:** Rehab Mahmoud, Hamdy F. M. Mohamed, Sarah H. M. Hafez, Yasser M. Gadelhak, E. E. Abdel-Hady

**Affiliations:** 1 Department of Chemistry, Faculty of Science, Beni-Suef University, P.O. Box 62514, Beni Suef, Egypt; 2 Physics Department, Faculty of Science, Minia University, P.O. Box 61519, Minya, Egypt; 3 Materials Science and Nanotechnology Department, Faculty of Postgraduate Studies for Advanced Sciences, Beni-Suef University, P.O. Box 62511, Beni Suef, Egypt

**Keywords:** Chemistry, Energy science and technology, Engineering, Materials science, Nanoscience and technology

## Abstract

Finding suitable non-expensive electrocatalyst materials for methanol oxidation is a significant challenge. Waste valorization of spent wastewater nanoadsorbents is a promising route toward achieving circular economy guidelines. In this study, the residual of layered double hydroxide (LDH) can be used as an electrocatalyst in direct methanol fuel cells as a novel approach. The Co–Ni–Zn–Fe LDH was prepared by the co-precipitation method followed by the adsorption of methyl orange (MO). Moreover, the spent adsorbent was calcined at different temperatures (200, 400, and 600 °C) to be converted to the corresponding mixed metal oxides (MMO). The prepared samples were characterized using XRD, FTIR, HRTEM, zeta potential, and hydrodynamic size measurements. The spent adsorbent was tested as an electro-catalyst for direct methanol electro-oxidation. The spent LDH/MO adsorbent showed a maximum current density of 6.66 mA/cm^2^ at a 50 mV/s scan rate and a 1 M methanol concentration. The spent MMO/MO adsorbent showed a maximum current density of 8.40 mA/cm^2^ at a 200 °C calcination temperature, 50 mV/s scan rate, and a 3 M methanol concentration. Both samples show reasonable stability over time, as indicated by the chronoamperometric response. Further nanoengineering of used nanoadsorbents could be a promising path to repurposing these wastes as electro-oxidation catalysts.

## Introduction

Producing energy in a clean and sustainable manner is one of the world’s major challenges. Fuel cells have attracted a lot of attention for power generation compared to other standard energy storage devices because of their unique characteristics such as low cost, low emissions innovative smog pollution, and simple structure^1^. Fuel cell technologies are a clean method for producing electrical energy from stored chemical energy with high direct conversion efficiency and little environmental pollution^2^. Direct methanol fuel cells (DMFCs) are promising power sources for applications such as electric vehicles and portable electronic devices. Low operating temperature, ease of transportation and fuel storage, high energy efficiency, and quick start-up are only a few of the benefits of methanol as a fuel^3^. Platinum is the most commonly used DMFC anode catalyst because it has an excellent electrocatalytic activity for methanol and long-term stability in an electrolyte solution. However, platinum is expensive and quickly poisoned by CO or other carbonaceous intermediates, resulting in a decrease in electrocatalytic performance^4,5^.

Because of their high surface area to volume ratio, unusual structure, and appealing physicochemical features, nanomaterials are interesting in the study of electrochemistry^6^. A significant number of atomically thin 2D materials have been synthesized, comprising metallic chalcogenides, phosphorene, boron nitride, and layered double hydroxides (LDHs)^7^. LDHs are primarily made up of positively charged brucite-like metallic hydroxide layers separated by weakly bonded intercalating hydrated anions, which are commonly denoted as [M_(1−x)_^2+^M_(x)_^3+^(OH)_2_]^x+^[A^n−^]_x/n_·mH_2_O, where M^2+^ and M^3+^ are divalent and trivalent metal cations, respectively, and A^n−^ is the charge-balancing anion^8^. The residual of LDH can be used as an electrocatalyst in direct methanol fuel cells (DMFCs) as a novel approach^9^. Waste valorization has been recognized as one of the optimal routes toward achieving and implementing the strategies and stages of a circular economy^10^. Waste valorization offers a sustainable approach to mitigating the adverse environmental impacts of municipal and industrial wastes^11^. One of the challenging sources of industrial waste is spent nanoadsorbents saturated with various types of pollutants. The main source of water pollution is the organic dyes released from industrial wastes which are extremely harmful due to their carcinogenic nature^12^. Several methods for removing biological and water pollutants from the environment and household wastewater have been developed^13^. Exploring new routes for reusing spent adsorbents is a persistent and challenging task that requires considerable effort to tackle. Rial et al.^14^. Recently reviewed the reported approaches to valorizing spent wastewater adsorbents. Such approaches include recycling them as catalysts for chemical synthesis, re-applying them as further adsorbents for other pollutants, their usage as cementitious fillers, and/or their usage in some miscellaneous industrial applications^14^.

Our research group has contributed to this research track by exploring novel techniques to reuse and/or valorize spent adsorbents. Moustafa et al.^15^ reported the successful reuse of spent Zn–Fe layered double hydroxide (LDH) nanoadsorbents after being used for methyl orange (MO) adsorption as methylene blue (MB) adsorbents. The authors reported a fast adsorption equilibrium time of 5 min and attributed MB adsorption to the new functional groups created upon the introduction of MO to the Zn–Fe LDH surface. Mahmoud et al.^16^ used Zn–Fe-LDH for dye adsorption in single and ternary systems (methyl orange, malachite blue, and methylene green). They investigated the structure of the prepared material using physical and chemical methods. The adsorption mechanisms of dyes were studied using XRD and FTIR analyses, as well as Monte Carlo simulation. LDH also demonstrated that it could be used as a photocatalyst for dye-polluted water. Abdel-Hady et al.^9^ followed a different approach where Zn–Co–Fe LDH was used as a nanoadsorbent for MB adsorption. To follow, the spent adsorbent was tested as a direct methanol electro-oxidation catalyst. The authors reported a maximum current density of 41.11 mA/cm^2^ at 50 mV/s and a 3 M methanol concentration.

In this study, we report reusing double substituted Co–Ni–Zn–Fe LDH spent adsorbent, after being used for MO adsorption, as an electro-catalyst for direct methanol electro-oxidation. Moreover, the spent LDH adsorbent were calcined at different temperatures (200, 400, and 600 °C) to convert the samples to the corresponding two-dimensional mixed metal oxides (MMO). All samples were tested as direct methanol fuel cell (DMFC) anodes in a three-electrode electrochemical setup at different methanol concentrations and scan rates. The use of residual LDHs to modify electrodes is a novel technique for catalyzing methanol oxidation.

## Materials and methods

### Materials

Zinc nitrate and nickel nitrate were purchased from Chem-Lab NV (Belgium) and Alpha chemical (India), respectively. Both Ferric nitrate and cobalt nitrate were supplied by Oxford (India). Hydrochloric acid was supplied by Carlo Erba reagents (France), while NaOH was obtained from Piochem for laboratory chemicals (Egypt). Methyl orange (MO) was purchased from Oxford Laboratory Reagents (India). All the chemicals used were analytical reagent grade and they were used with no further purification (as they were received).

### Synthesis of Co–Ni–Zn–Fe LDH

**Figure 1:**
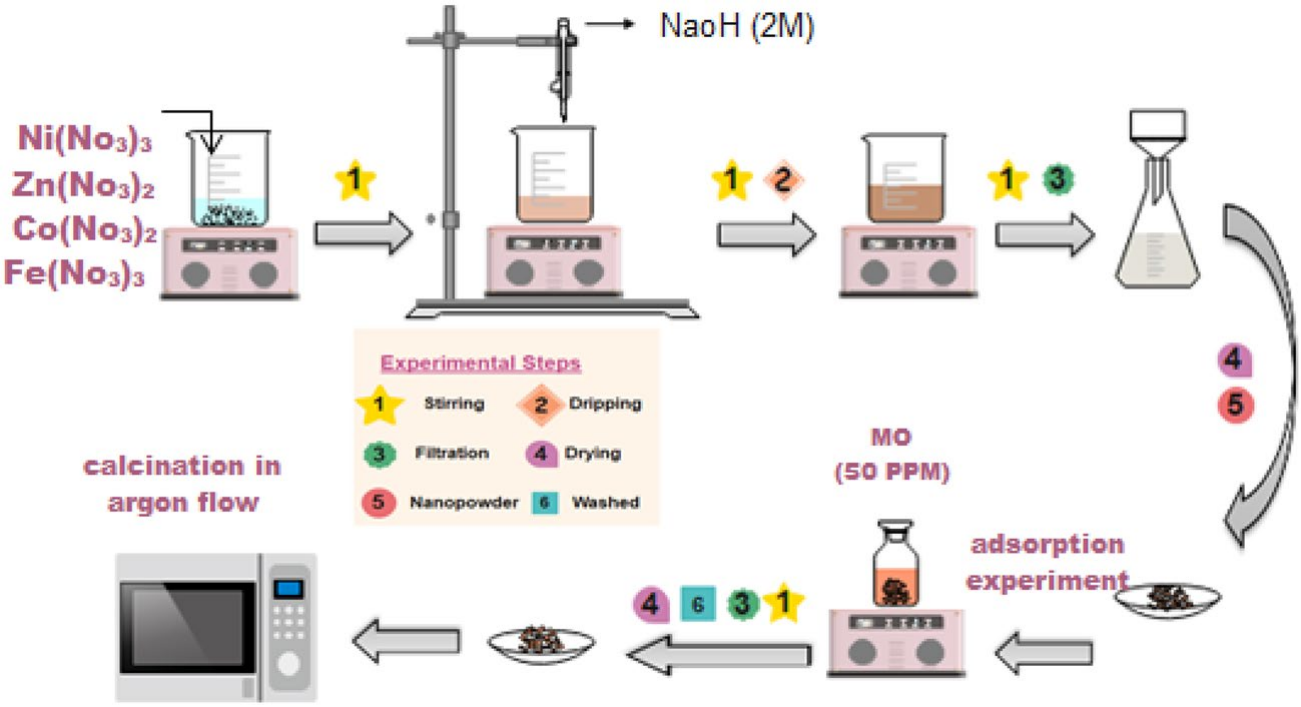
Preparation of Co–Ni–Zn–Fe LDH and Co–Ni–Zn–Fe LDH/MO.

The co-precipitation method was used to prepare Co–Ni substituted Zn-Fe LDH following a procedure similar to our previous work^15^. The co-precipitation method is used to give a crystalline size in the small range compared to other synthesis processes with an extremely homogeneous mixture of cations^17,18^. Figure 1 shows the preparation of Co–Ni–Zn–Fe LDH. Briefly, the metal nitrates were mixed in a 1:1:1:1 molar ratio and precipitated using the slow addition (0.1 mL/min) of 2 M NaOH solution until the pH of the solution was reached 8 to guaranty complete precipitation. The resulting material was aged overnight under continuous stirring. The formed suspension was then filtered and washed several times using distilled water to get rid of excess OH and then washed using ethanol. From fluency samples before and after adsorption were calcined at 200, 400, and 600 °C in an inert atmosphere (Argon flow). Figure 1 was produced using Wondershare EdrawMax Version 10 software.

### Synthesis of Co–Ni–Zn–Fe LDH/MO

0.05 g Co–Ni–Zn–Fe LDH was added to falcon tube containing 50 mL of MO diluted solution (50 ppm). The falcon tube was shaken using an orbital shaker (SO330-Pro) for 24 h at 250 rpm until reaching equilibrium. After the adsorption experiment, filtration (using Millipore Millex-G, 0.22 µm pore size syringe filters) was used to separate the catalyst from the solution. Then, washed many times to obtain the Co–Ni–Zn–Fe LDH/MO nanocomposite, which was dried at 50 °C for 12 h^15^.

### Zeta potential evaluation

The zeta potential and hydrodynamic particle size are used to investigate charges on the surface of a substance in solution and to quantify the magnitude of electrostatic attractions, aggregation causes, particle stability, and average particle size^19^. The synthetic samples were thoroughly dispersed in deionized water under ultrasonication to produce a suspended colloid with a concentration of 1.0 g/L. Before the measurements, the temperature was set to 25 °C. The test is carried out in a pH-neutral environment (pH = 7)^20^.

### Characterizations of the prepared material

The synthesized LDH was characterized by XRD (PANalytical Empyean, Sweden). The accelerating voltage used was 40 kV, and 30 mA current (ranging from 5 to 60° scan angle and a scan step of 0.05°). Bruker (vertex 70 FTIR-FT Raman), Germany FTIR spectrophotometry (serial number 1341) covering a frequency range of 400—4000 cm^−1^ was used for FTIR measurements. Samples were applied using KBr discs. Zeta potential and hydrodynamic particle size were investigated by the nanozeta sizer (Malvern Instruments Ltd, United Kingdom). The procedure of sample preparation for zeta potential measurements was as explained in our previous work^21^. The microstructures of the prepared LDH were determined using high-resolution transmission electron microscopy (HRTEM, JEOL, JEM-2100, Tokyo, Japan).

### Methanol electro-oxidation experiments

#### Preparation of the working electrode

The glassy carbon (GC) electrode, which has a geometric area of 0.031 cm^2^ (with the diameter of 2 mm and have around shape) was cleaned until it was mirror-like, and then washed with distilled water and acetone before use. To prepare the working electrode, 5 mg of the produced composite was dispersed in a solution containing 400 μL isopropanol and 50 μL Nafion (D-521 dispersion, 5% w/w in water and 1-propanol, ≥ 0.92 meq/g exchange—Alfa Aesar), then treated with a powerful ultrasonic for 30 min at room temperature (25 °C). 10 μL of the prepared solution was dripped several times onto the active area of the GC electrode. Finally, the GC electrode was dried at 40 °C before the electrochemical tests^10^.

#### Electrocatalytic activity measurements

Electrochemical measurements were managed by versaSTAT 3, AMETEK, connected to a 3-electrode system with a reference electrode (Ag/AgCl), a counter electrode (Pt wire), and a working electrode (GC electrode covered by the prepared samples). Different concentrations of methanol from 0.5 to 3 M dissolved in a solution of 1 M KOH have been used. Cyclic voltammetry (CV) was measured over the potential window between 0 and 0.6 V, and with a scanning rate of 50 mV/s. The current densities were calculated based on the actual geometry of the glassy carbon electrode's surface area (0.031 cm^2^).

## Results and discussion

### Material characterization

**Figure 2:**
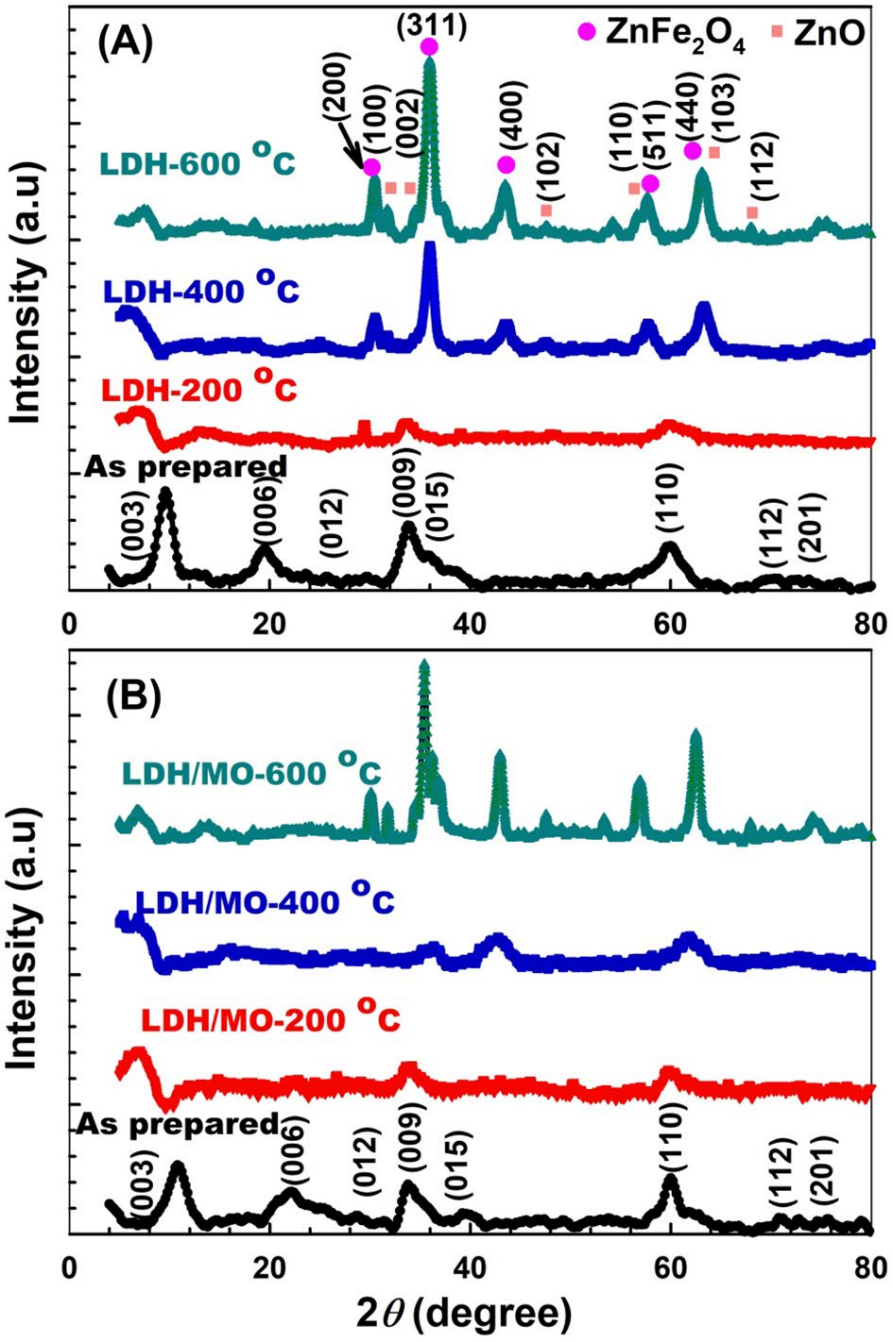
XRD of LDH samples (**A**) before and (**B**) after adsorption of MO.

The X-ray diffractograms of the samples before and after adsorption are shown in Fig. 2. The as-prepared LDH sample (Fig. 2A) showed typical peaks of the layered double hydroxide phase. The reflection peaks at 9.61°, 19.56°, 25.5°, 34.36°, 35.92°, 59.77°, 69.76°, and 73.6° correspond to plane families (003), (006), (012), (009), (015), (110), (112), and (201), respectively^22^. As shown in Fig. 2B, for the as prepared LDH sample after adsorption (LDH/MO), the intensity of some diffraction peaks decreased. This could be attributed to the adsorption of MO on the LDH surface, resulting in the diminishing of the diffraction intensity resulting from certain planes. The LDH/MO (2θ = 10.76°) basal plane corresponds to an interlayer distance of (8.21 Å), which is less than that of the as prepared LDH (9.61  Å). This is a strong indication that the MO is adsorbed on the LDH layers but not intercalated between the layers^23^. It is believed that when the dye is adsorbed on the surface, it causes the layers of the LDH phase to squeeze or pack closer together thereby the interlayer distance decreases slightly. On the other hand if the MO dye had been intercalated inside the layers, the interlayer distance should have increased which did not occur for the LDH sample after adsorption.

As also shown in Fig. 2A, after calcination, the LDH phase transformed into the corresponding mixed metal oxide (MMO) where both zinc oxide (ZnO) and zinc ferrite (ZnFe_2_O_4_) phases are formed. The peaks at 31.8°, 34.5°, 47.6°, 56.6°, 62.9°, and 68° correspond to the (100), (002), (102), (110), (103), and (112) planes of ZnO, respectively^24^. On the other hand, the peaks at 30.4°, 36°, 43.3°, 57.6°, and 63.2° correspond to the (200), (311), (400), (511), and (440) planes of ZnFe_2_O_4_ , respectively^25^. Calcination of the spent adsorbent samples did not correspond to any observable changes in the X-ray diffraction pattern, compared to the LDH samples before adsorption, as shown in Fig. 2B.

“Table 1 lists the crystallite sizes for the highest peak intensity at 2θ = 35.96° for the prepared nanocomposites calculated using Deby-Sherrer's formula^26^. It is clear from the table that, for the as-prepared LDH sample, the crystallite size of LDH (2.77 nm) is smaller than that of the LDH/MO (4.59 nm). Similar trend was observed for all the calcined samples where the crystallite size slightly increased for the samples after adsorption as compared to those before adsorption. It may be assumed that the dye molecules adsorbed on the surface of the LDH phase slightly assist in the preservance of the long-range order of the LDH and the MMO phase formed after calcination. On the other hand a significant increase in the crystallite size after adsorption for the sample calcined at 600 °C (19.82 nm) compared to that before adsorption (8.16 nm) could be observed (as shown in Table 2). This increase may be attributed to the local heating caused by the thermal oxidation of the adsorbed dye molecules at such high temperatures, which may cause improvement in the long-range order of the formed MMO phase. It is apparent that the crystallite size of the LDH or LDH/MO samples at calcination 200 °C is the least when compared with calcination at 600 °C. There is a strong influence of the electrocatalyst’s crystallite size on the activity of methanol oxidation.

**Table 1:** The crystallite size of the prepared LDH and LDH/MO samples.

Samples	Crystallite size (nm)	
	Before adsorption	After adsorption
As prepared	2.77	4.59
LDH-calcination 200 °C	3.45	4.17
LDH-calcination 400 °C	5.65	6.92
LDH-calcination 600 °C	8.16	19.82

**Table 2:** Zeta potential and particle size of prepared LDHs.

	Before the adsorption of MO		After the adsorption of MO	
	Zeta potential (mV)	Zeta sizer (nm)	Zeta potential (mV)	Zeta sizer (nm)
LDH-200 °C	27	331	30	964
LDH-400 °C	15	229	18	336
LDH-600 °C	5	2693	14	531

**Figure 3:**
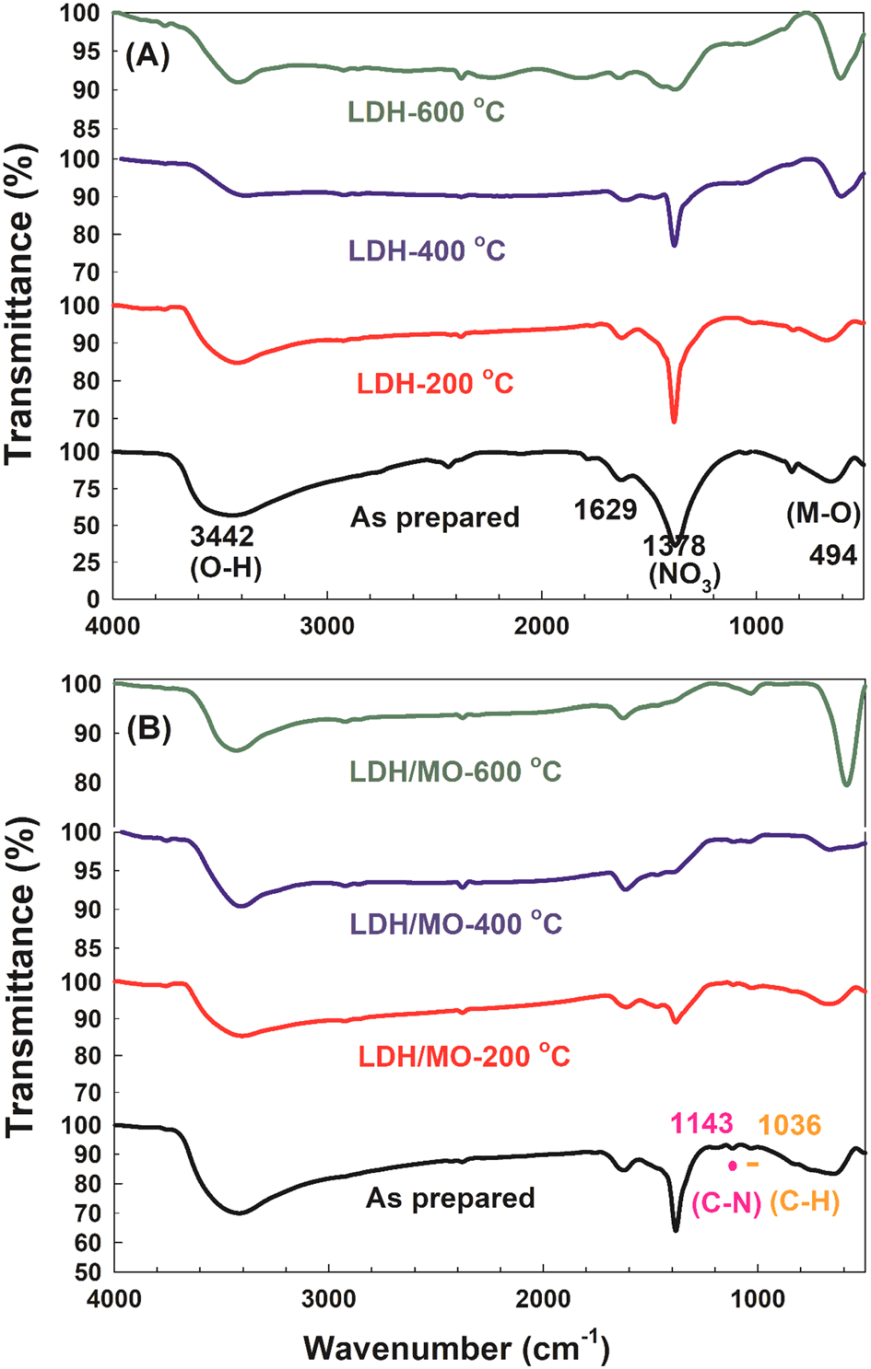
FTIR of LDH samples (**A**) before and (**B**) after adsorption of MO.

The FTIR spectra of the LDH samples before and after the adsorption of MO are shown in Fig. 3. As shown in Fig. 3A, the band around 3442 cm^−1^ could be ascribed to the characteristic free OH-stretching of the LDH structure vibration with the interlayer water molecules and hydrogen bonding^27^. On the other hand, the band at 1629 cm^−1^ is due to the bending vibration of the interlayer H_2_O water molecules^28,29^. The band at 1378 cm^−1^ was attributed to the ν_3_ stretching vibration of the nitrate groups in the interlayer of LDH^30^. This band intensity decreased with calcination temperature elevation due to the decomposition of nitrates into NO and/or any residual carbonate into CO_2_ with increasing temperature as reported before^31,32^. Below 1000 cm^−1^, the observed band resulted from the metal oxide (O–Me–O, Me–O, Me–O–Me) vibrations in the clay-like layers^33,34^. On the other hand, as shown in Fig. 3B, two new bands appeared in the spectrum of LDH after adsorption of MO. The first band at 1143 cm^−1^ is due to the bending vibrations of the C–N functional groups of MO, while the second band at 1036 cm^−1^ is attributed to the C–H bending vibrations of MO heterocycle^35^.

**Figure 4:**
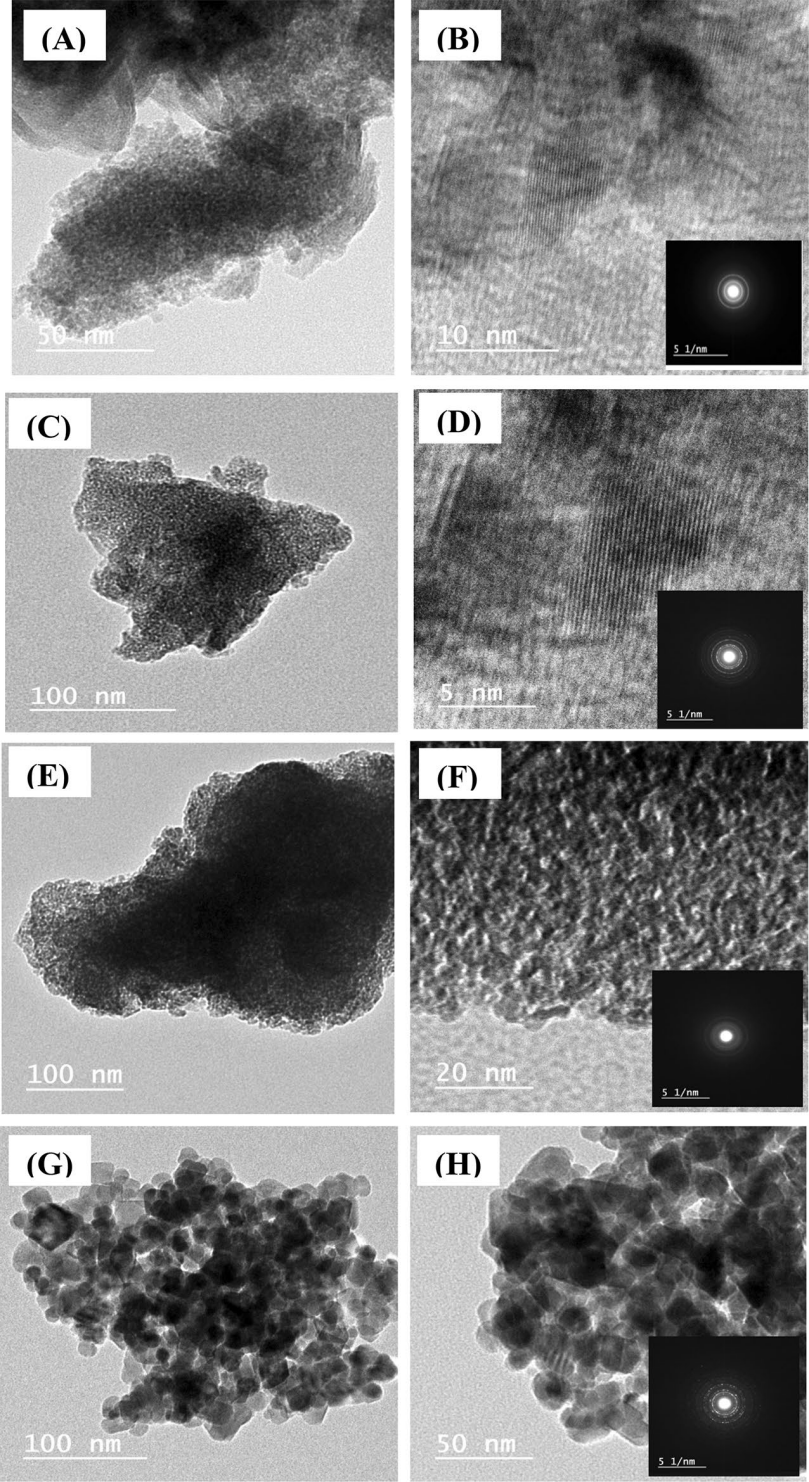
HRTEM images of as-prepared LDH sample (**A**, **B**); HRTEM images of LDH sample after adsorption at different calcination temperatures, 200 °C (**C**, **D**), 400 °C (**E**, **F**), and 600 °C (**G**, **H**). Insets are the corresponding SAED patterns.

Transmission electron microscopy (TEM) was used to further describe the microstructure of the synthesis of LDH nanosheets. Figure 4A showed the HRTEM image of the prepared LDH sample, which illustrates the hierarchical and porous dense-packing nanosheets, clearly demonstrating a layered LDH structure. It is clear from the image that no observed nanoclusters or nanoparticles are on the surface of the LDH nanolayers. On the other hand, the selected area electron diffraction (SAED) pattern reflects the semi-crystalline nature of the LDH phase due to the presence of concentric rings as presented in insets of Fig. 4B, in addition, similar to a plate, hexagonal and uniform structure in nature^36^. Figure 4C–H shows HRTEM images for the samples after adsorption at different calcination temperatures. Figure 4C,D shows the sample calcined at 200 °C, the calcined sample has transformed into the platelets particles confirmed the perfect lamellar structure, preserving the 2D layered morphology of the parent LDH. In the case of the sample calcined at 400 °C (shown in Fig. 4E,F), its observed that, the disappearance of the platelets like particles of LDH and appearance of agglomerates. The darker lines indicate the presence of aggregate crystallites which probably obtained from a dense agglomeration of LDH particles. By increasing the calcination temperature to 600 °C (shown in Fig. 4G,H) causes crystallinity and size to rise. The crystallinity of the calcined samples was much improved for the sample calcined at 600 °C as evident from the SAED pattern in the inset of Fig. 4H which is in a good agreement with the XRD results. It can be concluded that higher calcination temperatures favour long-range order of the atoms thereby increasing the crystallite size as evident by the SAED pattern and XRD results (as discussed in Table 1). On the other hand, higher temperature breaks the as prepared layers into aggregated pieces. Similar results were reported for the calcination of MgAl LDH at 500 °C, which transformed into aggregated "nanoflakes" or platelets compared to the original layered structure of the parent LDH^37^.

**Figure 5:**
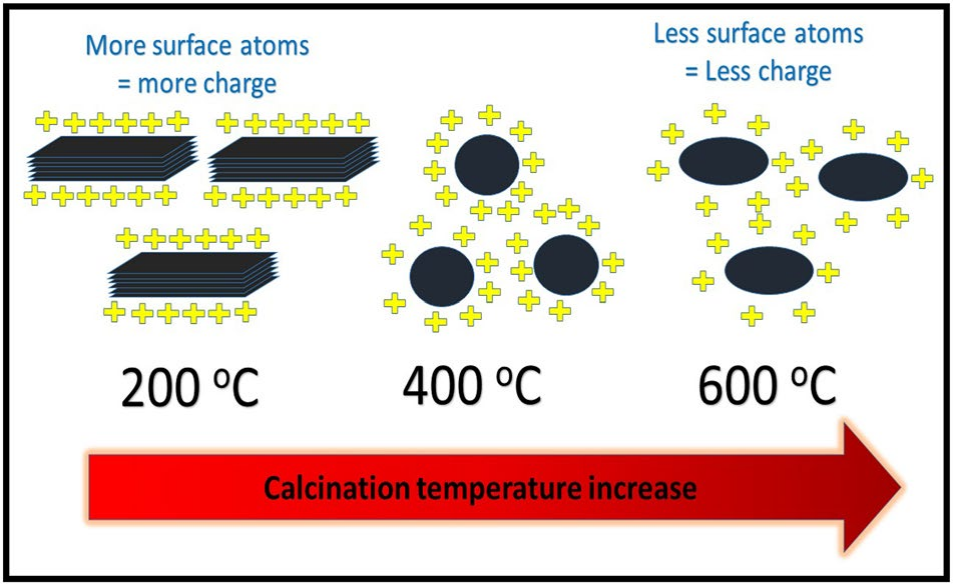
Explanation of the mechanism of zeta potential decrease with calcination temperature.

The Zeta potential and particle size of the prepared LDHs at different calcination temperatures are listed in Table 2. As shown in Table 2, with the increase in calcination temperature the zeta potential decreased. Samples calcined at 200, 400, and 600 °C showed a zeta potential of 27, 15, and 5 mV, respectively. This can be explained based on the change of particle morphology after calcination as discussed in the previous section. With calcination temperature increase, particles change from platelet like to agglomerates to larger agglomerates. This change in morphology can change the ratio of surface atoms to body atoms. More atoms will be inside the formed particles with the increase in temperature thereby surface atoms will decrease. With the decrease in surface atoms the overall unbalanced charges on the surface atoms decrease thereby the zeta potential decreases. This is further explained in the following schematic diagram (Fig. 5). Similar findings were reported by Li et al.^38^ for ceria octahedrons where the calcination temperature decreased the zeta potential of the samples.

As shown in Table 2, for the sample calcined at 200 °C, the zeta potential was estimated to be 27 mV. This value slightly increased for the sample after adsorption calcined at the same temperature. Similarly, the sample calcined at 400 °C showed a slight increase from 15 mV before MO adsorption to 18 mV for the sample after adsorption. On the other hand, the sample calcined at 600 °C showed a different behavior. Before adsorption, the estimated zeta potential was 5 mV while after adsorption the zeta potential was 14 mV. It seems that this behavior can be related to the crystallite sizes demonstrated in Table 1. It can be assumed that the long-range order detected from XRD pattern (as previously discussed) can be reflected in the values of zeta potential. It can be assumed that the long-range order can preserve the positive charge of the layers in MMO phase after calcination. On the other hand, short-term order seems to decrease the overall charge on the surface of the MMO layers. This is because amorphous (or semi-crystalline) domains on the MMO layer can deteriorate the electric field originating from surface atoms thereby decreasing the zeta potential. Electric filed from one of these domains can cancel or decrease that of the adjacent or nearby fields because of the amorphous nature of atom ordering. On the other hand, long-range order enhances the electric field on the surface atoms and enhances the overall surface charge.

The hydrodynamic particle size is illustrated in Table 2. With the decrease in zeta potential, hydrodynamic sizes increase due to the decrease in electrostatic repulsion between particles leading to agglomeration. However, for the calcined MMO samples the hydrodynamic size change did not correspond solely to the effect of zeta potential. It seems that other factors affect the degree of agglomeration such as the possible fusion and stickiness of the particles originating from the calcination of the LDH layers as discussed in the TEM images.

### Recycling the LDH as a methanol electro-oxidation catalyst

**Figure 6:**
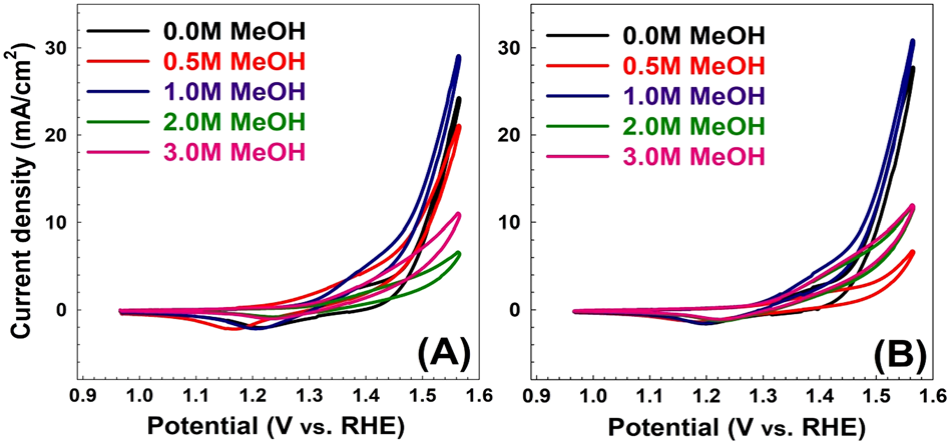
Cyclic voltammetry of as prepared samples (**A**) before and (**B**) after MO adsorption for different methanol oxidation at 50 mV/S scan rate.

To investigate the reuse of the spent adsorbent, the performance of the LDH/MO samples was tested as methanol oxidation electro-catalyst at different initial methanol concentrations. Moreover, the samples were calcined at different calcination temperatures, thereby resulting in the corresponding mixed metal oxides (MMO), which were also studied. The cyclic voltammetry (CV) results of prepared LDH samples before and after adsorption are shown in Fig. 6. The measured potentials vs. Ag/AgCl were converted to the reversible hydrogen electrode (RHE) scale according to the Nernst equation:

$${\text{E}}_{{{\text{RHE}}}} = {\text{E}}_{{{\text{Ag}}/{\text{AgCl}}}} + {\text{E}}^{{\text{o}}}_{{{\text{Ag}}/{\text{AgCl}}}} + 0.0{\text{59 pH}}$$

where E_RHE_ is the converted potential vs. RHE, E_Ag/AgCl_ is the experimentally measured potential against Ag/AgCl reference, and E^°^_Ag/AgCl_ = 0.1976 at 25 °C^39^. Electrochemical activities of LDHs were acquired at an applied potential of 0–0.6 V (vs. Ag/AgCl) in 1 M KOH recorded.

The electrochemical activity of the LDH (Fig. 6A) and LDH/MO (Fig. 6B) were studied in an aqueous electrolyte on 1M KOH at different methanol concentrations and at a scan rate of 50 mV/s. The current density at the end of the anodic peak for LDH at 0, 0.5, 1, 2, and 3M methanol was 24.33, 21.26, 28.8, 6.26, and 10.68 mA/cm^2^, respectively. The current density at the end of the anodic peak for the spent LDH/MO adsorbent at 0, 0.5, 1, 2, and 3M methanol was 27.30, 6.39, 31.15, 11.86, and 11.88 mA/cm^2^, respectively. This interesting enhancement in current density can be attributed to successful methanol oxidation. Therefore, more electrons can be produced^40^. Additionally, the current density of the oxidizing peak for LDH at 0, 0.5, 1, 2, and 3M methanol was 3.06, 4.92, 6.53, 2.91, and 4.20 mA/cm^2^, respectively. The current density of the oxidizing peak for the spent LDH/MO adsorbent at 0, 0.5, 1, 2, and 3M methanol is 3.45, 2.51, 6.66, 4.65, and 5.18 mA/cm^2^, respectively. The data states that the LDH after adsorption almost has a two-fold current density increase compared to that of LDH before adsorption at 2M methanol concentration. Methanol concentration augmentation leads to a reaction rate increase due to the diffusion phenomena and methanol concentration in the LDH layer, which leads to better oxidation of methanol. However, when the concentration of methanol is low, adequate methanol does not reach LDH layer, so it suffers more concentration loss^41^.

Some relevant works by LDHs are summarized in Table 3. From this table, the maximum current density after adsorption at 3 M methanol is comparable to that reported in the open literature for Ni–Cr LDH at 60 mV/s and the same methanol concentration, which showed a maximum value of 7.02 mA/cm^2^^42^. On the other hand, the measured maximum current density is lower than that reported by Abdel-Hady et al.^9^ for the spent Zn–Co–Fe LDH saturated with methylene blue (MB). The authors reported a maximum current density of 2.29, 4.61, 11.97, and 12.61 mA/cm^2^ for 0, 0.5, 1, 2, and 3 M methanol concentration, respectively, at a scan rate of 50 mV/s. This indicates that further nanoengineering of the spent catalyst is a promising route to enhance its performance for methanol oxidation.

**Table 3:** Comparison of this work with several reported works on catalytic activity.

Catalyst	Electrolyte	Scan rate (mV/s)	Current density	References
NiCr/LDH	1.0 M KOH	60	7.02 mA/cm^2^	^42^
	3 M CH_3_OH			
Zn–Co–Fe LDH/MB	1.0 M KOH	50	12.61 mA/cm^2^	^9^
	3 M CH_3_OH			
Pt black	1.0 M NaOH - 1.0 M CH_3_OH	100	83.6 mA mg^−1^ Pt	^43^
Commercial Pt/C	1.0 M NaOH - 1.0 M CH_3_OH	100	107.3 mA mg^−1^ Pt	^43^
Pt/Ni–Fe LDH	1.0 M NaOH - 1.0 M CH_3_OH	100	119.2 mA mg^−1^ Pt	^43^
Pt/C	1.0 M KOH - 1.0 M C_2_H_5_OH	50	6.0 mA/cm^2^	^44^
Pd/C	1.0 M KOH- 1.0 M C_2_H_5_OH	50	11.1 mA/cm^2^	^44^
Pt–CeO_2_	1.0 M KOH - 1.0 M C_2_H_5_OH	50	11.6 mA/cm^2^	^44^
Pd–CeO_2_	1.0 M KOH - 1.0 M C_2_H_5_OH	50	27.1 mA/cm^2^	^44^
Pt/C	0.1 M KOH- 1.0 M CH_3_OH	50	0.32 mA/cm^2^	^45^
Fe/N/C	0.1 M KOH - 1.0 M CH_3_OH	50	1.91 mA/cm^2^	^45^
Pt/STC	1.0 M H_2_SO_4_ - 1.0 M CH_3_OH	30	2.30 mA/cm^2^	^46^
Pt @ GO-PVP NPs	0.5 M H_2_SO_4_ - 0.5 M CH_3_OH	50	43 mA/cm^2^	^47^
Pt/C	0.5 M KOH - 0.5 M CH_3_OH	25	0.09 mA cm^−2^Pt	^48^
Pt–Ag/C	0.5 M KOH - 0.5 M CH_3_OH	25	0.16 mA cm^−2^Pt	^48^
Pt–Ag/SnO_2_-C	0.5 M KOH - 0.5 M CH_3_OH	25	0.21 mA cm^−2^Pt	^48^
rGO/PEDOT : PSS	0.5 M NaOH - 0.5 M CH_3_OH	50	37.5 mA/cm^2^	^49^

**Figure 7:**
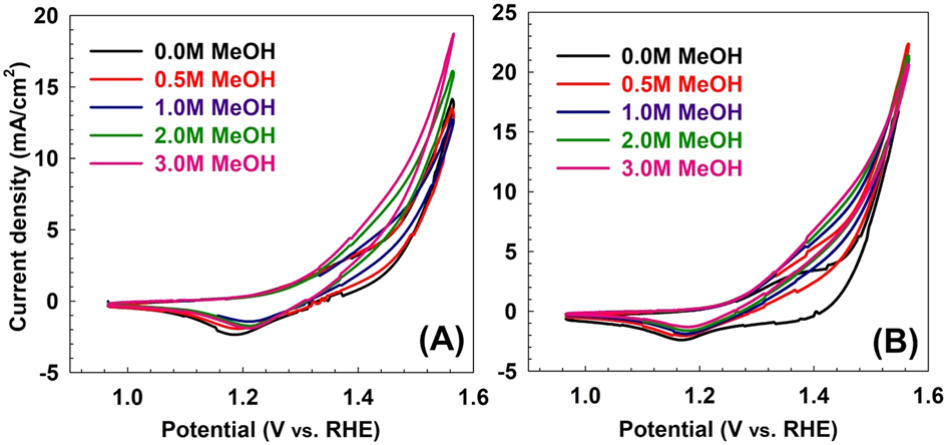
Cyclic voltammetry of samples calcined at 200 °C (**A**) before and (**B**) after MO adsorption for different methanol oxidation at 50 mV/s scan rate.

**Figure 8:**
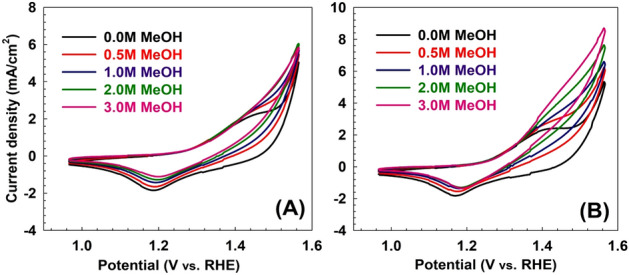
Cyclic voltammetry of samples calcined at 400 °C (**A**) before and (**B**) after MO adsorption for different methanol oxidation at 50 mV/s scan rate.

**Figure 9:**
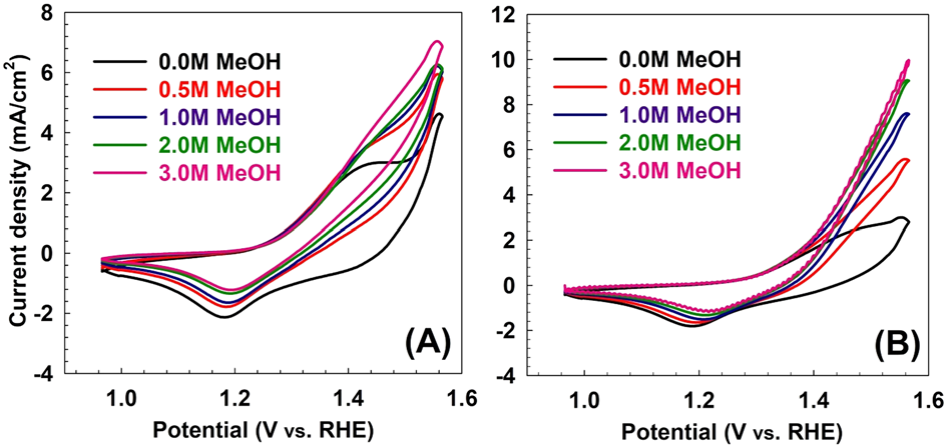
Cyclic voltammetry of samples calcined at 600 °C (**A**) before and (**B**) after MO adsorption for different methanol oxidation at 50 mV/s scan rate.

One of the possible strategies is to calcine the spent catalyst and study its performance at different calcination temperatures. This strategy was followed and the results for the performance of the calcined samples are discussed below. The CV results of LDH samples before and after adsorption at different calcination temperatures (200, 400, and 600 °C) are shown in Figs. 7, 8, 9. As shown in Fig. 7, the LDH sample calcined at 200 °C after adsorption showed that the current density at the end of the anodic peak was increased from 16.72 to 21.23 mA/cm^2^ as the methanol concentration increased from 0.0 to 3 M. On the other hand, the current densities of the oxidation peaks are changed from 5.98 to 8.40 mA/cm^2^ as the methanol concentration increased from 0.5 to 3 M. As shown in Fig. 8, for the sample calcined at 400 °C after adsorption, the current density at the end of the anodic peak was increased from 5.32 to 8.69 mA/cm^2^ as the methanol concentration increased from 0.0 to 3 M. On the other hand, the current densities of the oxidation peaks are changed from 2.90 to 4.19 mA/cm^2^ as the methanol concentration increased from 0.5 to 3 M. Similarly, as shown in Fig. 9, for the sample calcined at 600 °C after adsorption, the current density at the end of the anodic peak was increased from 3.04 to 9.69 mA/cm^2^ as the methanol concentration increased from 0.0 to 3 M. On the other hand, the current densities of the oxidation peaks are changed from 3.43 to 5.04 mA/cm^2^ as the methanol concentration increased from 0.5 to 3 M.

**Figure 10:**
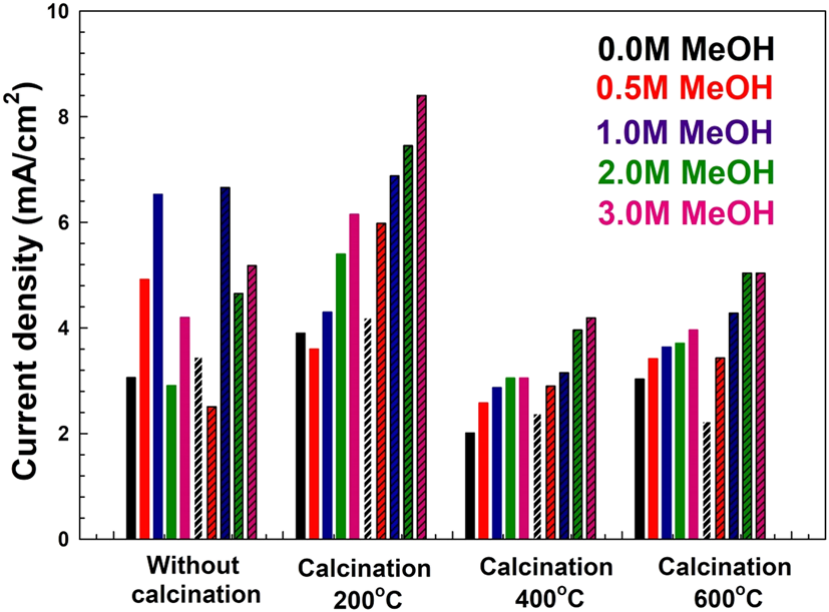
Summary of the current density and fold increases between before adsorption (bulk colour) and after adsorption (fine dashed colour) for all prepared samples.

Generally, further increasing the calcination temperature to 400 and 600 °C did not correspond to any further improvements in the value of the maximum current density. The corresponding maximum current density reached for each sample at each methanol concentration is summarized in Fig. 10. The sample after adsorption at calcination 200 °C showed a maximum current density of about 1.00–1.66 folds that of the sample before adsorption for all methanol concentrations. The maximum enhancement was for 1 M initial methanol concentration leading to a current density of 6.66 mA/cm^2^. On the other hand, the maximum current density for the calcined sample after adsorption was observed at 3 M methanol with a value of 8.40 mA/cm^2^.

**Figure 11:**
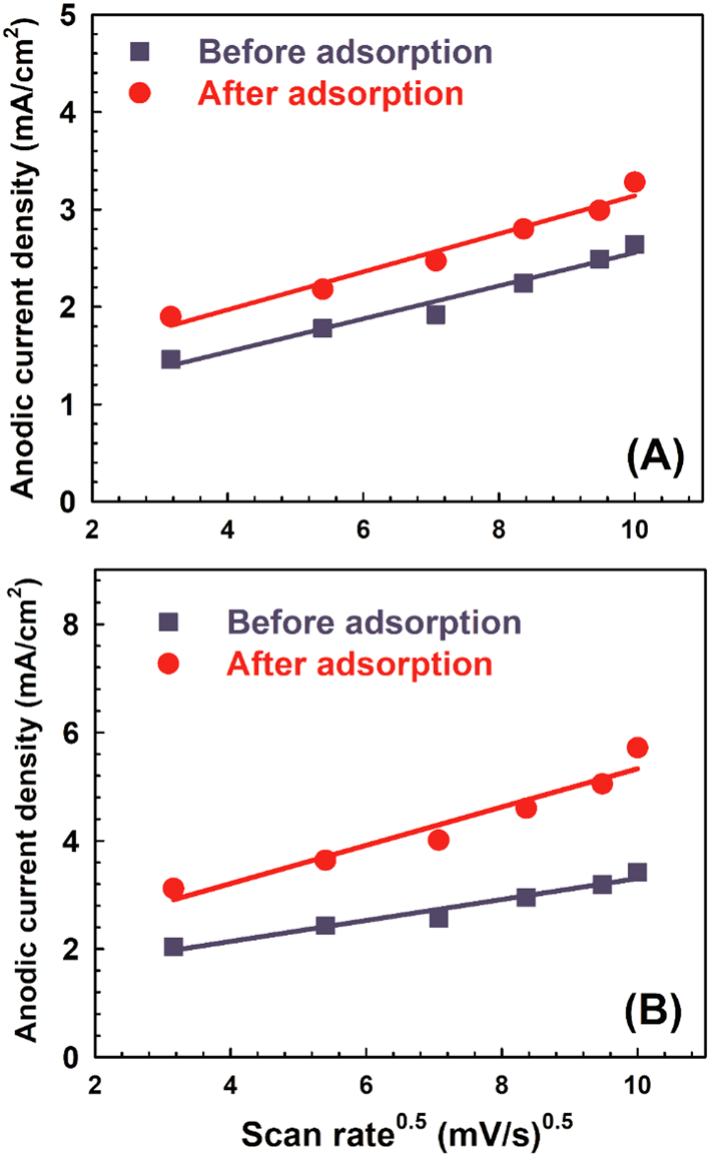
The relationship between the peak current density and the square root of the scan rates before and after adsorption for (**A**) LDH without calcination and (**B**) sample calcined at 200 °C under optimum conditions.

Figure 11 shows the relationship between the anodic peak current density and the square root of the scan rates (10–100 mV/s) for LDH before and after adsorption for LDH without calcination and the sample calcined at 200 °C at optimum conditions. It is clear from the figure that it is a linear relationship, which suggests that the electro-catalytic oxidation of methanol is a diffusion-controlled process^50^. Changing the scan rate results in an increase in redox reactions, as seen in Fig. 11, and the electrochemical process is controlled by the propagation of OH in the mesopores. Table 4 illustrates the slope and correlation coefficient for the samples' anodic current densities (I_a_). The sample that was calcined at 200 °C after adsorption has a larger line slope than the other samples. This difference in slope is the result of the different electrocatalytic capabilities and demonstrates the higher level of electrochemical activity.

**Table 4:** The slope and correlation factor in the anodic current densities (*I*_a_).

Parameters	LDH without calcination		LDH-200 °C	
	Before adsorption	After adsorption	Before adsorption	After adsorption
Slope	0.86	1.18	1.36	1.79
R^2^	0.97	0.97	0.96	0.94

**Figure 12:**
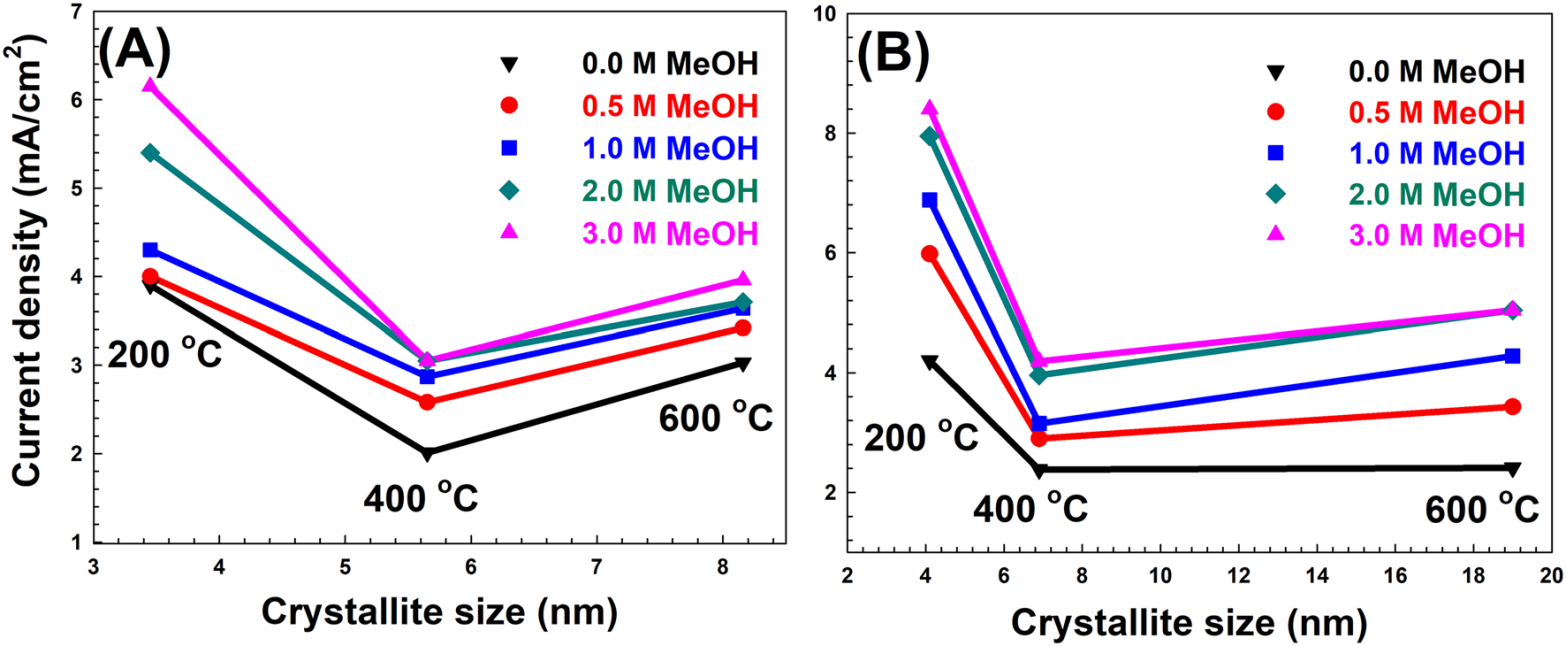
The maximum current densities obtained for the calcined samples versus the crystallite sizes for the samples (A) before and (B) after MO adsorption.

The data states that the LDH after adsorption almost has a two-fold current density increase compared to that of LDH before adsorption at 2 M methanol concentration. The enhancement of the activity for the MO adsorbed sample might be attributed to the positive interaction between the dye molecules and methanol. Such interaction might facilitate the diffusion of methanol molecules from the bulk of solution to the LDH layers where the active transition metal octahedron centres are present. As discussed earlier (in Fig. 11), the methanol oxidation process on the samples is a diffusion controlled process and aiding the mass transfer of methanol molecules may have a positive effect on enhancing the measured electrochemical activity. Figure 12 shows the maximum current densities obtained for the calcined samples versus the crystallite sizes. As shown in Fig. 12A, generally higher activities were recorded for samples with least crystallite sizes. Similar trend was observed for the samples after adsorption (Fig. 12B).

**Figure 13:**
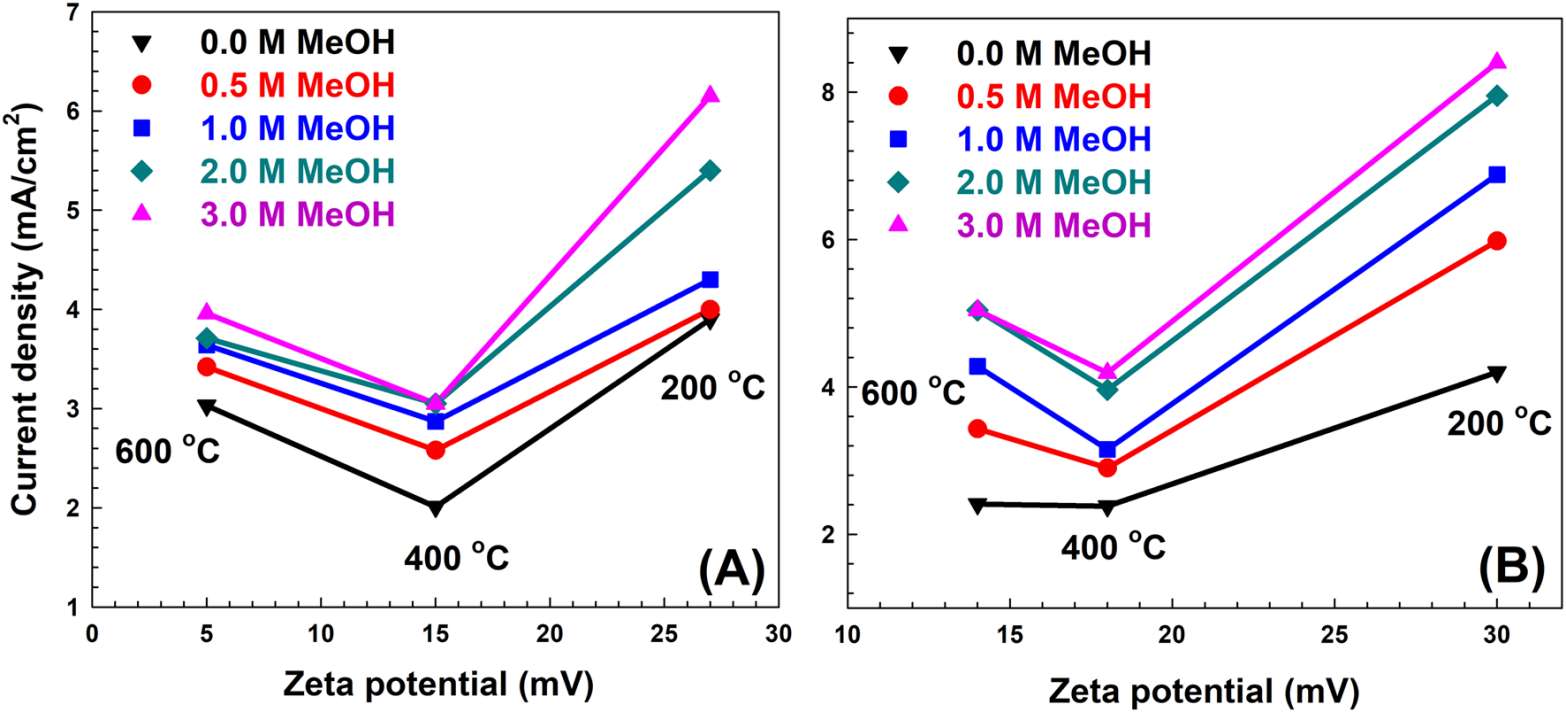
The maximum current densities obtained for the calcined samples versus the zeta potential for the samples (A) before and (B) after MO adsorption﻿.

Figure 13 shows the maximum current densities obtained for the calcined samples versus the zeta potential. It can concluded that higher activities were obtained generally for samples with the highest zeta potential either before or after adsorption as illustrated in Fig. 13A, B, respectively. It can be inferred from Figs. 12 and 13 that both crystallite size and zeta potential play a significant role in the value of the measured activity. Methanol oxidation process was demonstrated to be a diffusion controlled process (as discussed before), therefore small crystallite sizes may favour easier access of methanol molecules to the catalytic sites on the MMO layer surface. In addition, positive zeta potential for the anode material would facilitate the oxidation of methanol molecule while losing the electrons to the counter electrode. However describing the exact mechanism for methanol electro-oxidation over the surface of the calcined MMO is a challenging task. Wang et al.^51^ discussed the mechanism of methanol electro-oxidation over NiAl LDH films and discussed the complicated nature of the oxidation mechanism. Wang et al. supported the oxyhydroxide mechanism where Ni hydroxide in the LDH layers is converted into NiOOH species, which plays an important role in methanol oxidation. The prepared samples contains Ni, Co, Zn and Fe hydroxides, which may have different roles in the measured activities towards methanol oxidation in this study. Moreover, the carbon atoms produced after calcination of the samples, which is conducted in inert atmosphere, might have a role in altering the electric conductivity of the electrode material and its interaction with methanol molecules. All these factors might have roles with various degrees of significance to contribute towards the measured activity for the samples before and after adsorption. An exact mechanism describing the detailed primary reaction steps for methanol oxidation over the sample is considered a debatable issue, which might require further detailed studies to confirm.

**Figure 14:**
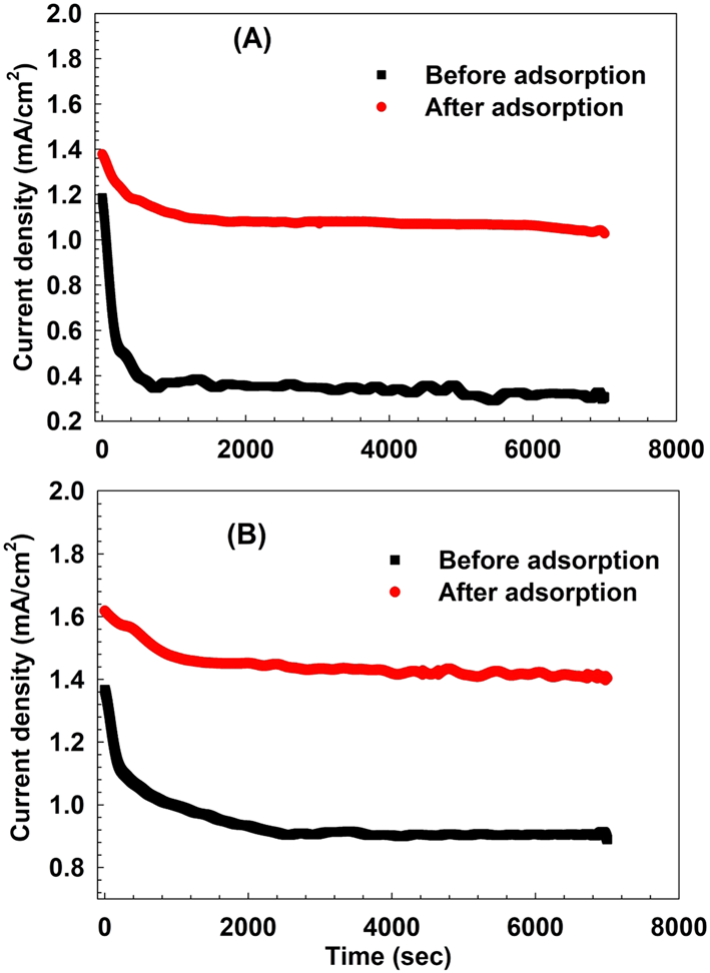
Chronoamperometric response of (**A**) LDH without calcination and (**B**) sample calcined at 200 °C under optimum conditions.

Moreover, Chronoamperometry was used to further evaluate the electrocatalytic activity and stability of the samples in 1 M KOH. The current density curves versus time were measured at a potential step of 600 mV for 7000 s as shown in Fig. 14. Initially, the current densities of the LDHs before and after adsorption decreased rapidly due to the double layer contribution, and then slowly decreased as time passed, which is due to the formation of the carbonaceous intermediate CO_ads_ on surface-active sites^52^. All samples show reasonable stability over time, where the current density of the samples did not decrease with increasing reaction time, indicating good electrocatalytic stability.

## Conclusions

In this work, Co and Ni double substituted Zn–Fe LDH layered double hydroxide was successfully prepared using a simple co-precipitation technique. The sample was used as a wastewater nanoadsorbent for MO adsorption. The spent adsorbent was successfully reused as a methanol electro-oxidation catalyst where results indicated an enhancement of performance compared to the freshly prepared samples without adsorption. Moreover, the samples were calcinated at 200, 400, and 600 °C to yield the corresponding mixed metal oxides. Cyclic voltammetry results indicate that the samples, in general, retained their performance after adsorption for different methanol concentrations, as indicated by the values of maximum current density. Samples with maximum performance enhancement showed stable performance with time, as indicated by Chronoamperometric measurements. This study paves the road for the valorization of spent nanoadsorbents toward various important technological applications such as direct alcohol fuel cells. Therefore, further nanoengineering of spent nanoadsorbents can be a promising route toward the re-utilization of such waste as promising electro-oxidation catalysts.
